# Tender solitary lesion in vulvar lichen sclerosus

**DOI:** 10.1016/j.jdcr.2022.01.038

**Published:** 2022-03-09

**Authors:** Lina Saeed, Bonnie A. Lee, Christina N. Kraus

**Affiliations:** Department of Dermatology, University of California, Irvine, California

**Keywords:** case reports, lichen sclerosus, plasma cell vulvitis, vulvar, vulvar disease, LS, Lichen sclerosus, PCV, Plasma cell vulvitis

A 61-year-old woman with a history of lichen sclerosus (LS) presented with intermittent vulvar pruritus for several years and new-onset tenderness at the right side of the introitus lasting for 3 months. She was applying clobetasol 0.05% ointment to the vulva once weekly with no improvement. Vulvar examination revealed white, shiny plaques with textural and architectural changes. An isolated bright red, shiny patch on the superior aspect of the right side of the introitus was noted, which appeared eroded, but which was confirmed intact on examination (Fig 1). A punch biopsy of this area revealed a dense lichenoid lymphoplasmacytic infiltrate with numerous plasma cells in the papillary dermis (Fig 2).

**Fig 1:**
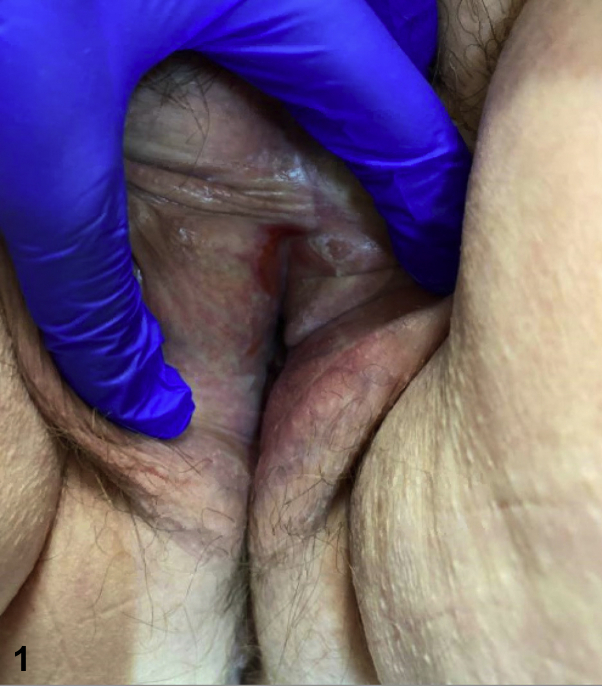

**Fig 2:**
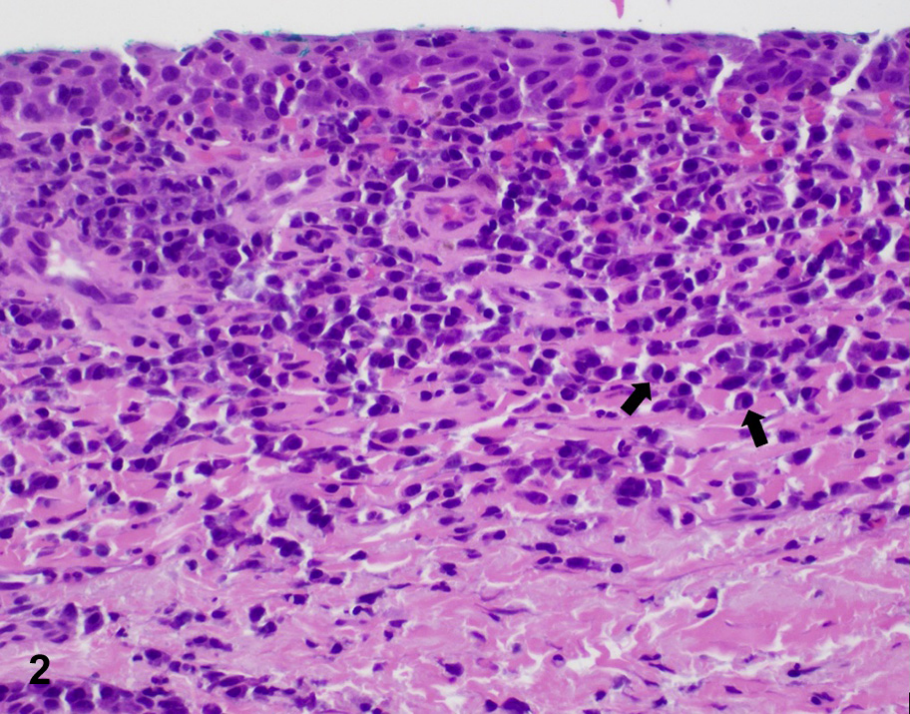

**Question 1: Which of the following is the best diagnosis for the introital lesion?**

A. Erosive lichen planus
B. Differentiated vulvar intraepithelial neoplasia
C. Pemphigus vulgaris
D. Plasma cell vulvitis (PCV)
E. Primary syphilis

**Answers:**

A. Erosive lichen planus – Incorrect. Erosive lichen planus is an immunologic condition that can present with a bright red solitary lesion; however, erosions are more common, and white lacy plaques may add in diagnosis. Histologically, erosive lichen planus would have a dense, lymphocytic lichenoid infiltrate, and while the infiltrate may contain plasma cells, plasma cells would not be the predominant feature.
B. Differentiated vulvar intraepithelial neoplasia – Incorrect. Differentiated vulvar intraepithelial neoplasia is a nonhuman papilloma virus-related precursor to vulvar squamous cell carcinoma. It commonly develops on a background of chronic inflammatory dermatoses, such as LS.^1^ While this could present as an isolated erythematous patch, pathologic examination would reveal full-thickness atypia, and sheets of plasma cells would not be seen.
C. Pemphigus vulgaris – Incorrect. Pemphigus vulgaris is an autoimmune intraepidermal bullous disease. While pemphigus can present with isolated red patches and erosions on the vulvar skin and mucosa, histopathologic analysis would reveal suprabasilar epidermal acantholysis.
D. PCV – Correct. PCV is an uncommon vulvar dermatosis which presents as bright red, orange, or brown well-demarcated macules/patches and pathologic examination reveals numerous plasma cells.^2^ It is thought that PCV develops in the setting of chronic inflammatory conditions,^3^^,^^4^ which may support its coexistence with LS, as in this case.
E. Primary syphilis – Incorrect. Syphilis is a sexually transmitted infection that may present on the vulva or vagina as a solitary tender lesion, usually ulcerated. Primary syphilis would not exhibit the chronicity reported in this case and is usually asymptomatic. While syphilis has similar histologic findings, including a lichenoid superficial and deep perivascular inflammation with admixed plasma cells, the clinical history make syphilis less likely.

**Question 2: Which of the following is the best first-line treatment modality?**

A. Excision with wide local margins
B. High-potency topical corticosteroids
C. Intramuscular penicillin
D. Mohs micrographic surgery
E. Topical imiquimod

**Answers:**

A. Excision with wide local margins – Incorrect. This is the treatment of choice for certain vulvar malignancies (squamous cell carcinoma, melanoma, etc.), but is not the treatment of choice for an inflammatory vulvar dermatosis.
B. High-potency topical corticosteroids – Correct. While comparative studies of most effective treatment modalities for PCV are limited, topical corticosteroids have the most evidence for symptomatic relief.^2^ Additionally, this patient had concomitant active LS, and high-potency topical corticosteroids are the treatment of choice. While this patient was using clobetasol ointment, she was only applying once a week, and the frequency for active LS or PCV should be daily.
C. Intramuscular penicillin – Incorrect. This would be the treatment of choice for primary syphilis.
D. Mohs micrographic surgery – Incorrect. This procedure may be considered for early squamous cell carcinoma or other early malignancies but is not the treatment of choice for an inflammatory vulvar dermatosis.
E. Topical imiquimod – Incorrect. Topical imiquimod is often used for genital warts and vulvar intraepithelial neoplasia. While the use of topical imiquimod has been reported for PCV, data are limited and inconclusive.^4^

**Question 3: Which of the following histopathologic findings would you NOT expect to see in this condition?**

A. Epidermal atrophy
B. Full-thickness atypia
C. Hemosiderin deposition
D. Spongiosis
E. Polyclonality

**Answers**:

A. Epidermal atrophy – Incorrect. Epidermal atrophy is a feature that can be observed in PCV.^2^
B. Full-thickness atypia – Correct. Full-thickness atypia is a feature associated with differentiated vulvar intraepithelial neoplasia and would not be seen in PCV. While there may be a reactive atypia associated with PCV, it would not be of the full-thickness type.^1^, ^2^
C. Hemosiderin deposition – Incorrect. Vascular proliferation with dilated vessels, hemosiderin deposition, and erythrocyte extravasation are findings observed in PCV.^2^
D. Spongiosis – Incorrect. Spongiosis is a feature commonly seen in PCV.^2^
E. Polyclonality – Incorrect. The plasmacytic infiltrates in PCV have been demonstrated to be polyclonal.^2^ This is in comparison to a monoclonal process, which would be seen in a condition like multiple myeloma.

## Conflicts of interest

None disclosed.
